# Cadaver-based hands-on course in cervical spine surgery: a prospective evaluation of surgical confidence and self-perceived autonomy

**DOI:** 10.1007/s00701-026-06939-8

**Published:** 2026-06-06

**Authors:** Aron Alakmeh, Victor Gabriel El-Hajj, Jonas Nordquist, Victor E. Staartjes, Erik Edström, Adrian Elmi-Terander

**Affiliations:** 1 Machine Intelligence in Clinical Neuroscience & Microsurgical Neuroanatomy (MICN) Laboratory, Department of Neurosurgery, Clinical Neuroscience Center, University Hospital Zurich, University of Zurich, Zurich, Switzerland; 2 Department of Clinical Neuroscience, Karolinska Institutet, Stockholm, Sweden; 3 Capio Spine Center Stockholm, Löwenströmska Hospital, Upplands-Väsby, Sweden; 4 Department of Medicine, Karolinska Institutet, Huddinge, Sweden; 5 Department of Medical Sciences, Örebro University, Örebro, Sweden; 6 Department of Surgical Sciences, Uppsala University, Uppsala, Sweden

**Keywords:** Teaching, Education, Cervical spine, Surgical navigation systems, Cervical surgery

## Abstract

**Purpose:**

Realistic off-patient training plays a crucial role in surgical education, particularly in complex and high-risk fields such as cervical spine surgery. To investigate effects of cadaver-based surgical education, this study investigates the educational value of a cadaver-based course in cervical spine surgery.

**Methods:**

24 participants completed a three-day cadaver-based training course covering anterior and posterior cervical spine approaches in March 2024 in Sweden. The course focused on anatomical dissection with supervised instrumentation. Participants completed pre-, post-course and 6-month follow-up questionnaires assessing prior experience, post-course satisfaction, and longitudinal changes in surgical confidence and clinical impact. Educational impact was analyzed based on Kirkpatrick’s model for evaluating training programs.

**Results:**

All respondents (*n* = 23/23, 100%) reported that the course met their expectations. At six-month follow-up, a considerable proportion of participants indicated increased surgical independence across all practiced procedures. The highest rates of perceived improvement (Kirkpatrick level 1 – reaction) in independent surgical performance were observed for anterior cervical discectomy and fusion (ACDF) procedures (*n* = 15/19, 78.9%) and laminectomies (*n* = 13/19, 68.4%). Cadaveric hands-on training (*n* = 13/19, 68.4%) and expert guidance (*n* = 3/19, 15.8%) were indicated as the most valued aspects of the course.

**Conclusion:**

This study highlights the perceived impact (Kirkpatrick level 1 – reaction) of a three-day hands-on course in cervical spine surgery, demonstrating its effectiveness in improving surgical confidence and self-perceived autonomy among participants, contributing to an increased number of independently performed procedures. Overall, cadaver-based training on anatomical specimens represents a valuable addition to surgical education in the operating room for cervical spine surgery.

## Introduction

Surgical training outside of operating on patients is a key part of surgical training in most surgical fields [8]. It enables the trainee to learn without any potential harm to a patient. Especially, as surgical procedures continue to rise in their complexity, effective yet safe training for surgical trainees is more needed than ever [21].

Various training modalities are available outside of the operating room, including surgery simulators in various modalities, virtual reality (VR) simulation, augmented reality (AR) simulation, synthetic anatomical models and lastly anatomical specimen models [17, 19, 23]. Among these modalities, the use of anatomical specimens, one of the earliest and most traditional methods, remain highly valued for their close approximation to intraoperative conditions [8]. This learning method has a high degree of authenticity and is hence considered to be of relevance to the learners which impacts their motivation and learning [15]. Despite known limitations of anatomical specimen models such as high cost, ethical considerations, exposure to toxic chemicals, biological waste and risk for disease transmission, cadaver-based training continues to play a central role in surgical education [2, 16]. This enduring relevance stems from its unmatched combination of tactile authenticity, exposure to the full spectrum of anatomical variation, and the spatial complexity encountered in real surgery – features that newer technologies like VR, AR, or synthetic models cannot fully replicate yet [4, 20]. This observation is also aligned with current educational theories [1, 10, 15].

The utility of training on anatomic specimens has been shown in various surgical subspecialties, especially in minimally invasive surgery, transplantation surgery, and in high-stake surgeries [9, 12, 13]. Reasons include the complexity of these procedures and limited teaching time in the OR due to the acuteness of the surgeries [9, 12, 13].

While positive effects of the use of anatomic specimen in surgical training in other surgical subspecialties, especially trauma surgery and vascular surgery, have been shown in various studies, there is a lack of studies on the effect of cadaver training in cervical spine surgery [9, 12, 22]. This is noteworthy, as cervical spine surgery is particularly suited for training on anatomical models. Reasons include the anatomical complexity of the spine and the potentially devastating consequences of surgical error in this region; ranging from irreversible neurological injury to permanent disability, and death [6, 18, 24]. This study evaluates the learning outcomes of a three-day hands-on, cadaver-based cervical spine surgery course by administering standardized questionnaires before, immediately after, and six months post-course, focusing on participants’ perceived skill development and confidence in the practiced procedures.

## Materials and methods

### Course information

Departing from Kirkpatrick’s model of evaluating training programs, this study evaluates the learning outcomes, of a three-day hands-on cadaver based cervical spine surgery course held in Sparsör, Sweden, on March 13–15, 2024 [14]. The course focused on both anterior and posterior surgical approaches to the cervical spine and was led by experienced neurosurgeons and orthopedic spine surgeons from prominent Swedish hospitals and academic institutions.

### Educational framing

The design of the three-day program was grounded in cognitive psychology, educational theory, and best practice in classroom teaching. From this perspective, there are three primary points of departure [1, 10, 15]. First, an educational program must build upon the participants’ prior experience and knowledge. All learners bring existing knowledge to a learning situation, which influences how they interpret and assess the relevance of new content. Inaccurate or incomplete prior knowledge can impede or distort understanding. This is particularly important when designing training for experienced professionals, such as practicing clinicians. It is also vital to consider that a group may be heterogeneous, with some participants lacking key foundational knowledge, while others may find that their established clinical routines and experiences inhibit the acquisition of new learning. Second, learners’ intrinsic motivation determines, directs, and sustains their engagement with learning. The perceived relevance of the content is crucial. Third, learning must be goal-oriented, and both instructors and learners need a clear understanding of the intended learning outcomes. This clarity is essential for providing timely, targeted feedback. General or unspecific feedback is insufficient. Numerous studies highlight the importance of feedback and formative assessment. In a widely cited study from 2008, Hattie demonstrated that feedback is pivotal for learning [10]. The author also stressed the importance of ensuring that learners not only receive feedback but also understand and act upon it.

Assessing the direct impact of an educational intervention is inherently complex, as it involves numerous confounding variables and lacks the controlled conditions typical of studies in the natural sciences. In 1980, Kirkpatrick introduced a model that remains widely used to assess educational outcomes [14]. He defines four levels of impact: reaction, learning, behavior, and results. Level 1 (reaction): this stage assesses participants’ initial impressions and overall satisfaction with the training, including their responses to the content, delivery, and the experience as a whole. Level 2 (learning): this level measures the knowledge, skills, or attitudes participants have gained, typically through tests, quizzes, or simulations. Level 3 (behavior): this level examines whether participants apply their learning in their professional context, assessing behavioral change through observations, feedback, or performance reviews. Level 4 (results): this level evaluates the broader organizational impact of the training, such as improvements in productivity, cost efficiency, service quality, or customer satisfaction.

Designed for both residents and attending surgeons in orthopedic spine surgery and neurosurgery, the course aimed to develop and refine surgical skills. The first day focused on introductions, lectures on relevant aspects of cervical anatomy and the surgical procedures to be performed during the course. The second day was dedicated to anterior cervical procedures, including anterior cervical discectomy and fusion (ACDF), anterior cervical corpectomy and fusion (ACCF), uncotomy (uncinectomy or uncinatectomy), corpectomy, and anterior dens screw placement. The third day focused on posterior techniques, including posterior foraminotomy, lateral mass screw placement (C3-C6), muscle-sparing laminectomy, pedicle screw placement at C2, C7 and T1, posterior fixation at C1-C2 level, and laminoplasty.

Each anatomical specimen was assigned to a fixed pair of participants, who remained with their designated specimen throughout the entire course to ensure continuity in anatomical orientation. To guarantee a high-quality learning environment, the course maintained a faculty-to-participant ratio of 1:4, allowing for close supervision, direct feedback, and individual guidance during each surgical step [1, 10, 15].

An instructor-based approach was used. All members of the faculty were experienced spine surgeons with more than 15 years of experience, and all worked at academic institutions. The total time for theoretical elements was 4 h, while 12 h were dedicated for practical education. All elements of each procedure were demonstrated on a large screen by a member of faculty, and the participants were guided by other members of faculty to follow step by step.

Surgical instruments for the procedures were provided by Medtronic (Minneapolis, MN, USA). Additional partners included PO-Medica (Stockholm, Sweden), Ethicon (Johnson & Johnson, New Brunswick, NJ, USA), Surgify (Helsinki, Finland), and Augmented Navigation (Stockholm, Sweden).

### Data acquisition

All 24 participants were asked to complete standardized questionnaires at three time points: prior to the start of the course, immediately after its completion, and six months following the course. These questionnaires intended to measure learners’ response to the educational intervention (Kirkpatrick level 1).

The pre-course questionnaire focused on assessing each participant’s baseline experience in cervical spine surgery. This is educationally important of three reasons. First, new knowledge is always built on previous knowledge and experience. Second, a pre-course evaluation activates the learner’s previous knowledge and experience. Third, the results of the pre-course evaluation enable the organizers to define expected learning outcomes for each participant. This is also significant importance for the experts to provide formative feedback during the program [1, 10]. Specifically, participants were asked whether they had previously assisted in or independently performed the included procedures in the course. In addition, they were queried about their previous use of surgical navigation during cervical procedures, their experience with intraoperative complications, the approximate number of cervical spine procedures they had independently performed – if any – and their individual expectations for the course.

The post-course questionnaire served two different purposes. The primary purpose was to measure the perceived educational impacts on Kirkpatrick level 1. A secondary purpose was to monitor the quality of the educational design and delivery of the course, The first part evaluated participant satisfaction (Kirkpatrick level 1) with various aspects of the course, including quality of instruction, intraoperative guidance, the learning environment, specimen quality, and the adequacy of provided surgical instruments. Each criterion was assessed using a five-point Likert scale, where a score of 1 represented the lowest possible rating and a score of 5 the highest. The second part included a series of binary (yes/no) questions aimed at identifying the participants’ overall perception of the course and potential areas for improvement (Kirkpatrick level 1).

Six months after course completion, participants were asked to reflect on how the course had impacted their clinical practice. They were asked whether their level of perceived independence in performing the practiced procedures had increased, remained unchanged, or decreased. Moreover, they were asked to evaluate their development in broader surgical competencies, including the use of navigation systems, operating microscopes, and the management of surgical complications. Finally, participants were asked to retrospectively comment on both positive and negative aspects of the course in order to improve the educational design and delivery for future editions of the course.

## Results

### Participants

A total of 24 participants attended the course, including 9 residents (37.5%) and 15 consultants (62.5%). Of these, 10 participants (41.7%) were trained or in training in neurosurgery, and 14 (58.3%) in orthopedic spine surgery. The median number of years in spine surgery was 5.0 (IQR: 2.0, 12.5). All 24 participants completed the pre- and post-course assessments; follow-up data at six months were available for 19 participants (Table 1).

**Table 1:** Summary of participant’s characteristics in the pre-course questionnaire

Parameter	Total (*n* = 24)
Residents, *n* (%)	9 (37.5)
Attendings, *n* (%)	12 (62.5)
Working in academic hospitals, *n* (%)	19 (79.2)
Working in private practice, *n* (%)	5 (20.8)
Trained in neurosurgery, *n* (%)	10 (41.7)
Trained in orthopedic surgery, *n* (%)	14 (58.3)
Median number of years in spine surgery [y], (median [IQR])	5 [2.0, 12.5]
Previous experience in assisting…, *n* (%)	
ACDF	23 (95.8)
ACCF	17 (70.8)
Laminectomy	22 (91.7)
Foraminotomy	14 (58.3)
Placement of lateral mass screws	18 (75.0)
Placement of pedicle screws	17 (70.8)
C1-C2 fixation	16 (66.7)
Previous experience in independently performing…, *n* (%)	
ACDF	11 (45.8)
ACCF	5 (20.8)
Laminectomy	16 (66.7)
Foraminotomy	5 (20.8)
Placement of lateral mass screws	9 (37.5)
Placement of pedicle screws	7 (29.2)
C1-C2 fixation	5 (20.8)
Previous experience in navigation for cervical procedures…, *n* (%)	12 (50.0)

*IQR* Interquartile range, *ACDF* Anterior Cervical Discectomy and Fusion, *ACCF* Anterior Cervical Corpectomy and Fusion

### Pre-course survey

Out of the 24 participants, between 14 (58.3%) and 23 (95.8%) had previous experience in assisting in cervical spine procedures, depending on the specific procedure. Independent surgical experience was lower: 16 (66.7%) had performed laminectomies, 11 (45.8%) ACDFs, 9 (37.5%) lateral mass screw placements, 7 (29.2%) pedicle screw insertions, and 5 (20.8%) ACCFs, C1–C2 fixations, or foraminotomies independently as the lead surgeon (Table 1).

### Immediate post-course survey

**Fig. 1:**
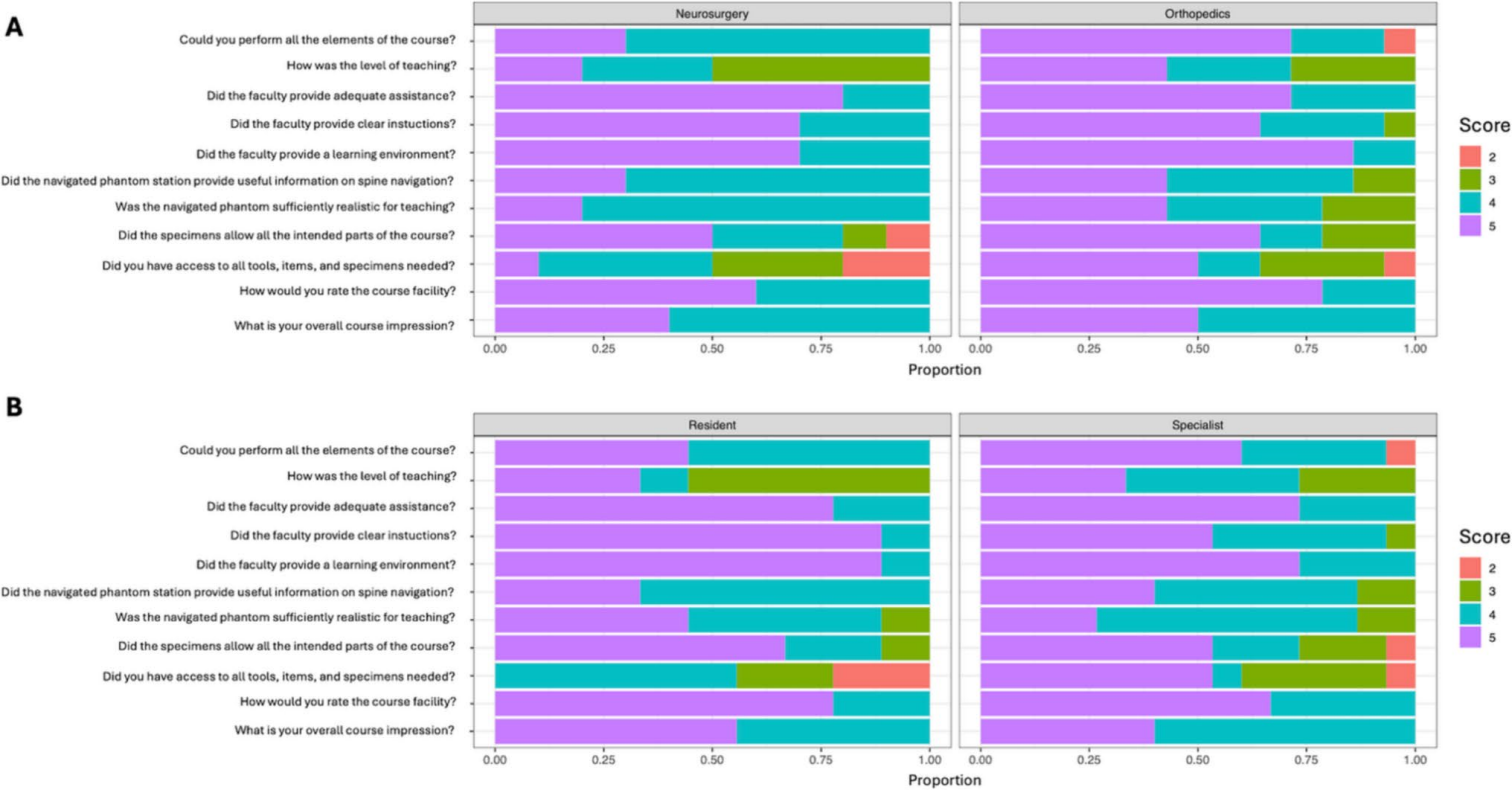
Post-course questionnaire responses on a 5-point likert scale

All 24 participants completed the post-course survey, though not all questions were answered by every participant. Overall satisfaction was high: all respondents confirmed the course met their expectations (20/20, 100%) and would recommend it to colleagues (23/23, 100%). 11 out of 13 respondents (84.6%) planned to modify their surgical technique based on the experience gained. Additionally, 10 out of 24 (41.7%) participants wished for more theory, and 9 out of 24 (37.5%) for more dissection time. On the 5-point Likert scale, ratings were consistently high as reported in detail in Table 2. However, access to instruments, materials, and specimen quality were identified as areas for improvement, with several participants assigning scores of 2 or 3. Notably, no one gave the lowest score (1) in any category (Fig. 1) (Table 2).

**Table 2:** Post-Course Questionnaire Responses

Parameter	Total (*n* = 24)
Adequate balance between theory and dissection time, *n* (%)	21 (87.5)
Desire for more theory, *n* (%)	10 (41.7)
Desire for more dissection time, *n* (%)	9 (37.5)
Course met expectations, *n* (%)	20/20* (100)
Would recommend course to colleagues, *n* (%)	23/23* (100)
Participants intending to implement learned skills in clinical practice, *n* (%)	11/14* (78.6)
5-point Likert Scale Rating, median (IQR)	
Course faculty	5.0 [4.0, 5.0]
Good access to tools, items and specimens	4.0 [3.0, 5.0]
Specimen allowing all the intended parts of the course	5.0 [4.0, 5.0]
Realism of navigation phantom	4.0 [4.0, 5.0]
Usefulness of navigation phantom station	4.0 [4.0, 5.0]
Learning environment	5.0 [5.0, 5.0]
Instruction clarity	5.0 [4.0, 5.0]
Adequate assistance provided by faculty	5.0 [4.0, 5.0]
Level of teaching	4.0 [3.0, 5.0]
Completion of all course elements	5.0 [4.0, 5.0]

*IQR* Interquartile range

*These questions were not answered by all 24 participants

### 6-month post-course survey

**Fig. 2:**
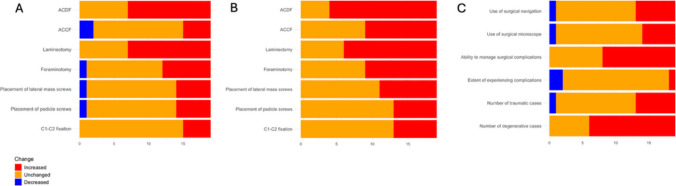
6-month post-course questionnaire responses showing changes in number of procedures performed (**A**), changes in independence in performing procedures (**B**), and changes in surgical experience (**C**). ACCF, Anterior Cervical Corpectomy and Fusion; ACDF, Anterior Cervical Discectomy and Fusion

19 of 24 participants (79.2%) completed the 6-month follow-up survey. Most respondents reported an increase in the frequency of ACDF, and laminectomy procedures performed (*n* = 12/19, 63.3%). For other procedures the proportion of respondents indicating an increase in frequency was relatively low. Confidence in managing surgical complications increased in 13 out of 19 respondents (68.4%). Increased use of microscopes was indicated by 5/19 respondents (26.3%) and increased use of navigation systems was indicated by 6/19 participants (31.6%). Perceived surgical independence rose in 6/19 (31.6%) to 15/19 (78.9%) of respondents depending on the procedures, with the highest independence gains for ACDF (*n* = 15/19, 78.9%) and lowest for pedicle screw/C1–C2 fixation (*n* = 6/19, 31.6%) (Fig. 2) (Table 3).

**Table 3:** 6-Month post-course questionnaire responses

Parameter (*n* = 24)	Increased	Unchanged	Decreased
Extent of experiencing complications, *n* (%)	1 (5.3%)	16 (84.2%)	2 (10.5%)
Ability to manage surgical complications, *n* (%)	11 (57.9%)	8 (42.1%)	0 (0.0%)
Use of surgical microscope, *n* (%)	5 (26.3%)	13 (68.4%)	1 (5.3%)
Use of surgical navigation, *n* (%)	6 (31.6%)	12 (63.2%)	1 (5.3%)
Number of procedures performed, *n* (%)			
ACDF, *n* (%)	12 (63.2%)	7 (36.8%)	0 (0.0%)
ACCF, *n* (%)	4 (21.1%)	13 (68.4%)	2 (10.5%)
Laminectomy, *n* (%)	12 (63.2%)	7 (36.8%)	0 (0.0%)
Foraminotomy, *n* (%)	7 (36.8%)	11 (57.9%)	1 (5.3%)
Placement of lateral mass screws, *n* (%)	5 (26.3%)	13 (68.4%)	1 (5.3%)
Placement of pedicle screws, *n* (%)	5 (26.3%)	13 (68.4%)	1 (5.3%)
C1-C2 fixation, *n* (%)	4 (21.1%)	15 (78.9%)	0 (0.0%)
Traumatic cases, *n* (%)	6 (31.6%)	12 (63.2%)	1 (5.3%)
Degenerative cases, *n* (%)	13 (68.4%)	6 (31.6%)	0 (0.0%)
Independence in performing, *n* (%)			
ACDF, *n* (%)	15 (78.9%)	4 (21.1%)	0 (0.0%)
ACCF, *n* (%)	10 (52.6%)	9 (47.4%)	0 (0.0%)
Laminectomy, *n* (%)	13 (68.4%)	6 (31.6%)	0 (0.0%)
Foraminotomy, *n* (%)	10 (52.6%)	9 (47.4%)	0 (0.0%)
Placement of lateral mass screws, *n* (%)	8 (42.1%)	11 (57.9%)	0 (0.0%)
Placement of pedicle screws, *n* (%)	6 (31.6%)	13 (68.4%)	0 (0.0%)
C1-C2 fixation, *n* (%)	6 (31.6%)	13 (68.4%)	0 (0.0%)

*ACDF* Anterior Cervical Discectomy and Fusion, *ACCF* Anterior Cervical Corpectomy and Fusion

The cadaver-based hands-on training was the most valued aspect of the course, cited by 11 out of 19 respondents (57.9%), followed by expert tips, selected by 4 respondents (21.1%). Twelve out of 19 (63.2%) respondents felt all topics received adequate attention; four out of 19 (21.1%) requested more theoretical and anatomical instruction. From a learning perspective, eight out of 19 (42.1%) respondents noted improved skills or confidence; one out of the 19 (5.3%) respondents indicated no benefit to their clinical practice (Fig. 2) (Table 3).

## Discussion

This study demonstrates the educational value of a hands-on cervical spine surgery course conducted on anatomical specimens. The findings show that the presented cadaver-based training course was perceived as highly effective in enhancing perceived surgical confidence, particularly in ACDFs and laminectomies. Additionally, it showed positive impact in perceived ability to manage surgical complications, thus fostering participants’ sense of independence in the operating room. Additionally, some participants reported increased use of surgical microscopes and navigation systems, although the majority indicated no change in their usage patterns. Participants who reported increased use of these techniques were likely less experienced, suggesting that the course enhanced their confidence and encouraged broader application in clinical practice. In contrast, more experienced surgeons demonstrated no notable change in usage, presumably due to pre-existing familiarity and routine integration of these modalities.

Notably, the reported increase in independently performed procedures closely paralleled the participants' perceived rise in confidence for those techniques. In particular, ACDF and laminectomies were performed more frequently following the course, aligning with the highest reported gains in perceived procedural confidence. This suggests that the course may have served as a steppingstone towards surgical prowess and independence. In this context, the degree to which participants’ clinical environments can provide learning opportunities is pivotal [3]. To foster continued skill development, participants must regularly perform procedures under appropriate supervision. Such opportunities must occur with adequate frequency and volume – an expectation formally embedded in the procedural requirements for graduation from most surgical residency programs [3]. In most clinical settings, ACDF and laminectomy procedures are much more common and may meet these criteria, while posterior fixations and C1-C2 fixations may not. This could be interpreted as a greater need for repetition courses on the less frequently performed procedures to compensate for the lack of clinical exposure.

The results align with prior work, demonstrating the benefits of cadaver-based surgical training in other high-stakes surgical disciplines, including trauma and vascular surgery [9, 12, 13]. While various simulation modalities – including synthetic models, VR and AR – play an increasing role in surgical education, cadaveric dissection continues to offer the most anatomically correct and realistic training environment, closely replicating the conditions encountered in actual surgery [4, 5, 7, 11, 20]. The findings of this study support the continued integration of cadaver-based surgical training into surgical education, given its substantial impact on participant confidence and the subsequent transfer of acquired skills into clinical practice.

Two areas for improvement in the course design consistently noted by participants were the limited availability of instruments and the quality of anatomical specimens. The latter represents an inherent challenge in cadaver-based training, as specimen quality is difficult to control. Variability in anatomical preservation and inter-individual anatomy may impair procedural reproducibility and limit the generalizability of the training experience. In contrast, limited instrument availability represents an easily modifiable factor that, given appropriate financial resources, can be readily addressed through improved logistical planning and institutional or industrial support. It is also notable that 42% (*n* = 10/24) of participants requested more theoretical background, suggesting that even in practical settings, theoretical framing remains essential for consolidating procedural knowledge.

From a learning perspective, the finding that 6 to 14 out of 19 (32% to 79%) participants reported an increased sense of independence six months post-course depending on the procedure, supports the educational value of cadaveric training. Moreover, the observation that surgical complication management and confidence in using microscopes and navigation systems also improved suggests that the benefits of such training extend beyond procedure-based technical skills and into broader operative competencies.

Although all participants, irrespective of prior surgical training in spine surgery, benefitted from the course, we believe that participants with basic surgical skills and prior exposure to cervical spine surgery have a higher learning potential. The possibility of transferring the obtained knowledge into clinical practice in close connection to the course will result in retaining the manual skills and may have a higher impact on practice changes.

### Limitations

Several limitations should be considered when interpreting the findings of this study. It is important to stress that educational impact has many different conceptualizations. This study departs from Kirkpatrick’s model and has mainly focused on the first level of educational impact, reaction. It is not possible to extrapolate whether new learning has been implemented into clinical practice (level 3) or how patients might benefit (level 4). First, the data are based on self-reported measures, including perceived procedural competence, confidence, and changes in surgical behavior, which are inherently prone to response bias and may not directly correlate with objective surgical performance or patient outcomes. Second, the lack of a control group limits the ability to differentiate the true effect of the cadaveric training from other concurrent educational or clinical experiences that participants may have had. Furthermore, the relatively small sample size (*n* = 24) reduces statistical power and generalizability but nonetheless reflects common practical limitations of hands-on cadaver-based courses. Additionally, some questions in the post-course questionnaire were not answered by all participants, and the 6-month post-course questionnaire was only completed by 19 out of the total 24 participants. This could potentially have introduced a response bias. Third, while a 6-month follow-up provides insight into mid-term outcomes, it remains uncertain whether observed gains in confidence and procedural activity are sustained in the longer term. Finally, although the course provided anatomical specimens enabling near-intraoperative conditions, factors such as anatomical degradation, tissue stiffness, and the absence of bleeding or intraoperative stress may limit the realism of cadaver-based training. Despite its limitations, this study offers a structured, evidence-informed approach to course design and delivery grounded in educational theory, contributing to the development of a stronger pedagogical culture in surgical training.

## Conclusion

This study highlights the perceived impact (Kirkpatrick level 1 – reaction) of a three-day hands-on course in cervical spine surgery, demonstrating its effectiveness in improving surgical confidence and self-perceived autonomy among participants, contributing to an increased number of independently performed procedures. The 6-month follow-up revealed the highest increase in confidence in ACDF and laminectomy procedures. The structured format, high faculty-to-participant ratio, and consistent use of a single specimen allowed participants to practice cervical spine procedures efficiently. Overall, cadaver-based training on anatomical specimens represents a valuable addition to surgical education in the operating room for cervical spine surgery.

## Data Availability

The data in support of our findings can be obtained upon reasonable request from the corresponding author.
